# Understanding the Effect of Deposition Technique on the Structure–Property Relationship of Polyaniline Thin Films Applied in Potentiometric pH Sensor

**DOI:** 10.3390/polym15163450

**Published:** 2023-08-18

**Authors:** Vinicius M. Fraga, Isabela T. Lovi, Luis M. G. Abegão, Hugo J. N. P. D. Mello

**Affiliations:** 1 Materials Physics Group, Physics Institute, Goiás Federal University, Samambaia Campus, Goiânia 74001-970, GO, Brazil; viniciusmota@discente.ufg.br (V.M.F.); isabelatalon@discente.ufg.br (I.T.L.); 2 Photonics Group, Physics Institute, Goiás Federal University, Samambaia Campus, Goiânia 74001-970, GO, Brazil

**Keywords:** conducting polymer, thin film, open circuit potential, sensitivity, structure–property relationship

## Abstract

The comprehension of potentiometric pH sensors with polymeric thin films for new and advanced applications is a constant technological need. The present study aimed to explore the relationship between the sensitivity and correlation coefficient of potentiometric pH sensors and the structure–property relationship of polyaniline thin films. The effect of the deposition method on the sample’s properties was evaluated. Galvanostatically electrodeposited and spin-coated polyaniline thin films were used as the sensing stage. Samples were electrodeposited with a current density of 0.5 mA/cm^2^ for 300, 600, and 1200 s and were spin coated for 60 s with an angular velocity of 500, 1000, and 2000 rpm. The electrodeposited set of films presented higher average sensitivity, 73.4 ± 1.3 mV/pH, compared to the spin-coated set, 59.2 ± 2.5 mV/pH. The electrodeposited films presented higher sensitivity due to their morphology, characterized by a larger roughness and thickness compared to spin-coated ones, favoring the potentiometric response. Also, their oxidation state, evaluated with cyclic voltammetry and UV-VIS spectroscopy, corroborates their sensing performance. The understanding of the structure–property relationship of the polymeric films affecting the pH detection is discussed based on the characteristics of the deposition method used.

## 1. Introduction

Generic polymers, also known as traditional or non-conductive polymers (NCPs), are characterized by their insulating nature and exhibit low electrical conductivity. NCPs comprise covalently bonded carbon-based repeating units (monomers) that could form long molecular chains—the absence of significant electronic delocalization in NCPs results in the lack of electrical conductivity. In contrast, conducting polymers (CPs) exhibit alternating single and double bonds along the polymer backbone [1], facilitating the delocalization of π-electrons, allowing CPs to have a high electrical conductivity while maintaining the characteristics of NCPs, such as flexibility and processability [2]. Moreover, the possibility of tailoring their properties by doping or oxidation/reduction processes, but also their light weight, flexibility, and cost-effectiveness, make them valuable for a wide range of applications in different fields of science and technology, such as electronics, energy storage, actuators, biotechnology, and sensing [3].

Polyaniline (PANI) is one of the most studied and applied CPs. Its electrical conductivity, electrochromism, processability, environmental stability, and tunable characteristics make it suitable for a broad range of applications in devices, such as supercapacitors, solar cells, diodes, organic transistors, chemical sensors, gas sensors, and biosensors [4,5,6,7,8]. PANI can exist in three theoretical oxidation states, which are defined by the ratio of amine to imine nitrogen atoms in its backbone. The leucoemeraldine (PANI-LE) is the fully reduced, pernigraniline (PANI-PE) is the fully oxidized, and emeraldine base (PANI-EB) is the half reduced/oxidized PANI [9]. The protonation (acid or electrochemical) of PANI structure causes changes in its properties, such as morphology [10], color [11], and conductivity [12]. Those changes in the PANI properties are the foundation of the chemical sensors [13,14,15]. A chemical sensor is a device designed to detect and measure the presence of specific chemical markers in the environment. The interaction of its sensing stage with the target analyte produces a measurable signal, after the signal conversion by the transduction stage, that can be quantified and correlated with its concentration [16].

PANI thin film can be used in sensors due to its high surface area and fast response time, compared to non-thin-film structures, two of the main requirements for sensing stages on chemical sensors [17,18]. The deposition of PANI thin films is the process of controlled and precise application of the material onto a substrate. Various chemical and physical techniques are available for the CP’s deposition process. For example, chemical vapor deposition (CVD), Langmuir–Blodgett (LB) technique, dip, drop, and spin coating are examples of chemical deposition processes. On the other hand, sputtering; thermal evaporation; and potentiostatic, potentiodynamic, and galvanostatic electrodepositions are physical deposition processes examples [19]. Each method offers its advantages in terms of film thickness, uniformity, adhesion, and scalability. The choice of deposition technique depends on the application and specific requirements of the film. Controlling the deposition parameters ensures PANI thin films with precise thickness, morphology, oxidation state, and tailored properties [20,21,22].

Potentiometric sensors are a subclass of the electrochemical sensors [23]. They measure the variation in the interfacial electric potential caused by the target analyte on the surface of a sensing electrode [24]. One important class of chemical potentiometric sensors is the one for pH detection. The pH sensors are relevant in numerous industries and scientific fields besides manufacturing, water treatment, agriculture, and diagnosis [25,26]. The search for real-time, accurate, efficient, and reliable determination of pH is still relevant. An option for potentiometric transduction is the one based on the measurement of the open circuit potential (OCP). OCP monitoring as the detection technique allows for real-time and accurate measurements [27,28]. The physical–chemical process for potentiometric pH determination in the OCP mode is described by the Nernst equation [29]:

(1) $$\Psi =\Psi_{0}-2.3\cdot \alpha \cdot \frac{RT}{F}\cdot pH,$$

in which Ψ is the measured electrical potential, $\Psi_{0}$ is the electrical potential at standard conditions, R is the ideal gas constant, T is the temperature, F is the Faraday constant, and α is a sensitivity factor considering the surface proton buffer capacity and the double-layer capacitance. The α parameter is proportional to the ability of the sensing layer surface to accumulate protons on its surface, given by the buffer capacity, and it is proportional to the number of surface sites [30]. The pH sensitivity is obtained from the electrical potential differential to the pH reaching the Nernst limit of 59.2 mV/pH when α approaches 1. Controlling the sensitivity of the potentiometric pH sensors can be achieved by structuring the sensing layer properly. Therefore, this work presents PANI thin films deposited by galvanostatic electrodeposition and spin coating techniques applied in potentiometric pH sensors as the sensing stage. The sample’s morphology and oxidation state were correlated with the sensor’s figures of merit. The deposition influences the structure–property relationship of the films, affecting the sensor’s performance.

## 2. Material and Methods

### 2.1. Materials

The fluorine-doped tin oxide (FTO) thin film deposited over a glass substrate (7 Ω/sq.), PANI (PANI-EB, M_W_ 50,000 g/mol), aniline (99.5%), and hydrous dibasic sodium phosphate (99%) were purchased from Sigma-Aldrich. The N,N-Dimethylformamide (DMF, P.A.) and hydrochloric acid (HCl, P.A.) were purchased from Neon, Brazil. The citric acid (99.5%) was obtained from Vetec, Brazil. All the chemicals were used as received without any further purification.

### 2.2. PANI Thin-Film Deposition

PANI thin films were prepared using two different techniques: galvanostatic electrodeposition and spin coating. FTO thin films were used as substrates after cleaning by ultrasonication (DI-water, ethanol, and acetone, 10 min each). The spin-coated PANI thin films were produced using a spin-coater G3P-8 (SCS), from a weight ratio of 1:100 polymer:solvent (DMF) solution. The PANI solution was stirred and ultrasonicated for 1 h each before use. The SC500, SC1000, and SC2000 samples were spin coated with a spin velocity of 500, 1000, and 2000 rpm, respectively, for 60 s. The final samples were annealed on a hot plate for 20 min at 120 °C.

The electrodeposited PANI thin films were produced using an AUTOLAB potentiostat (Metrohm, Herisau, Switzerland) in an aqueous polymerization solution with 0.1 mol/L aniline and 1.0 mol/L HCl. A conventional three-electrode electrochemical cell system was used. The FTO substrate was used as the working electrode. The reference electrode was an Ag|AgCl (3 mol/L) electrode, and a platinum foil was used as the counter electrode. All experiments were performed at room temperature. The J5T300, J5T600, and J5T1200 samples were electrodeposited with an applied current set as 0.75 mA for a current density of 0.5 mA/cm^2^ during 300, 600, and 1200 s, respectively. The six sensing films are shown in Figure S1.

### 2.3. Sample Characterization

A DektakXT stylus (Bruker, Billerica, MA, USA) profilometer was used to obtain the samples’ profiles for thickness and average surface roughness measurements. The recorded data were analyzed in the Mountains Map®10 (Digital Surf, Besançon, France) software. Scanning electron microscopy (SEM) characterization was performed in the samples using a JEOL JSM—6610 system with an operating voltage of 15–20 kV. Prior to the analysis, a 5 nm Au layer was sputtered over the samples to enhance the electrical conductivity. The images’ analysis was performed using ImageJ 1.52a (NIH) software. The potentiostat was used to obtain cyclic voltammograms (CVs) of the samples with a potential range from 0.2 V to 1.2 V at a scan rate of 50 mV/s. A Lambda 1050 WB (PerkinElmer, Waltham, MA, USA) spectrophotometer was used to record the samples’ UV-VIS absorbance spectra. The selected absorbance spectral window ranged from 350 to 850 nm with a 1.0 nm step.

### 2.4. Sensor Measurement

The potentiostat was used to measure the OCP continuously between the sensing samples and the Ag|AgCl reference electrode. The PANI thin films were kept immersed in the electrolytes for 60 s while the system was recording the potential. The films were carefully washed in distilled water between each measurement. McIlvaine buffer solutions, ranging from pH 2 to 8, were used as electrolytes to obtain the calibration curve of the pH sensor. The sensitivity (S) was obtained from the sensor’s calibration curve. The coefficient of determination (R^2^) was obtained from the fitted calibration curves, and it was used for sensor evaluation and for comparison with the literature. A linear regression fitting was performed as the data fulfilled the requirements for it, as can be seen in Figure S2. All experiments were performed in triplicate, and results are presented as a function of mean and standard deviation values.

## 3. Results and Discussion

The electrodeposited and spin-coated PANI thin films were evaluated according to their thickness, presented in Table 1. The thickness of the electrodeposited films increases with increasing the deposition time due to the deposited charge increase, in agreement with the theoretically predicted [31]. The J5T1200 film is the thickest electrodeposited sample with 121.5 ± 27.9 nm. The spin-coated films’ thickness decreases with increasing the angular velocity due to increased radial liquid flow, in agreement with the physical model [32]. The SC500 is the thickest spin-coated sample with 13.2 ± 1.1 nm. As expected, the chronopotentiometric curves for PANI thin-film fabrication show a constant induction time and deposition potential for the samples (see Figure S3 in the Supplementary Materials).

**Figure 1:**
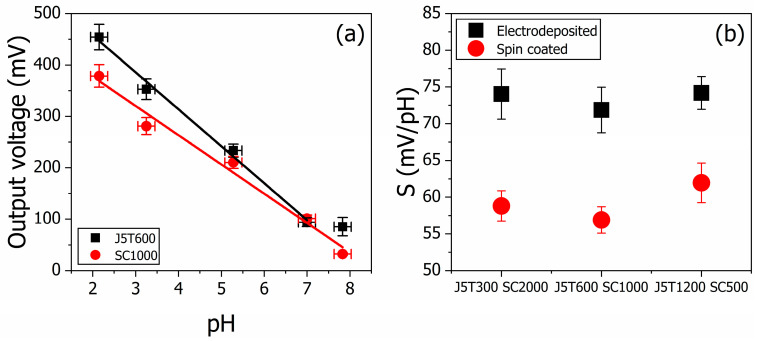
Potentiometric pH sensor response of electrodeposited and spin-coated PANI thin films. The output voltage response and the fitted calibration curves for the samples J5T600 and SC1000 are presented in (**a**). From the calibration curves, the devices’ sensitivity was calculated and presented in (**b**) for the two sets of PANI film. The linear fit regarding J5T600 (solid black line) does not consider the 7.8 pH once it seems the detection limit of the sensor. On the other hand, the linear fit of SC1000 (red solid line) is from 2.2 to 7.8 pH.

We performed the pH sensor measurements using a potentiometric platform with the PANI thin films. The output voltage response and the fitted calibration curves for samples J5T600 and SC1000 are presented in Figure 1a. Both curves were chosen as models of the device’s performance. The electrodeposited sample showed higher output voltage than the spin-coated one at each pH, as described elsewhere [33]. The sensitivity is also higher for J5T600 (71.9 ± 3.1 mV/pH) than for SC1000 (56.9 ± 1.8 mV/pH). The linear range for sample J5T600 (and for sample J5T300) is from pH 2.2 to 7.0, while for other samples, it is from pH 2.2 to 7.8. The PANI-based pH sensor is used in an acidic medium once the polymer does not show electroactivity in alkyl solutions [34], nor is it stable [35]. The response curves of the other films are not shown. The pH sensor works according to Nernst’s equation, indicating a decrease in the measured electrical potential with increasing the pH. The summary of the sensitivity analysis is depicted in Figure 1b. Two distinct groups indicate that the sensitivity of the potentiometric sensor is higher for electrodeposited samples than for spin-coated ones (average sensitivity of 73.4 ± 1.3 and 59.2 ± 2.5 mV/pH, respectively). The lower electrodeposited sensitivity is 71.9 ± 3.1 mV/pH for J5T600, and the higher one is 74.2 ± 2.2 mV/pH for J5T1200, while for the spin-coated set of films, it was 56.9 ± 1.8 mV/pH for SC1000 and 61.9 ± 2.7 mV/pH for SC500. The correlation coefficient of all samples was never lower than 0.97.

Notably, the electrodeposited and spin-coated PANI thin films demonstrated a remarkable advantage in terms of higher sensitivity when compared to other sensing materials employed in potentiometric sensors, as shown in Table 2. Table 2 assessed the performance of various potentiometric pH sensors through a comparative analysis including the material, sensitivity, deposition method, correlation coefficient, and substrate used. Lakard and co-workers [36] presented three polymeric thin films as pH sensors fabricated by electrodeposition over platinum (Pt) substrate. Polypyrrole (PPY), poly(p-phenylenediamine) (PPPD), and PANI were used, and the maximum sensitivity was 52 mV/pH for the PANI sample. This is lower than the sensitivity presented in this work for both deposition methods. Also, the electrodeposited PANI film presented higher sensitivity than three metal oxides materials, produced by different methods over different substrates, used for pH detection as iridium oxide (IrO_2_) [37], tungsten oxide nanoparticles (WO_3_ NP) [38], and titanium oxide (TiO_2_) [39]. Silicon nitride (Si_3_N_4_) thin films fabricated by low-pressure chemical vapor deposition (LPCVD) were also used as the sensing element for pH detection with a sensitivity of 49.7 mV/pH [40]. These results underscore the significance of our research and its potential applications in diverse fields, particularly where a high-sensitivity pH measurement is of utmost importance.

**Figure 2:**
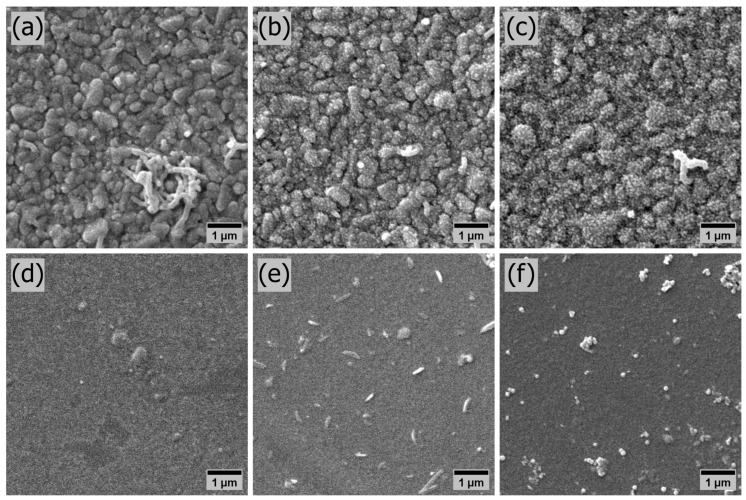
SEM characterization of the surface morphology of electrodeposited and spin-coated PANI thin films. The micrographs were enlarged 10,000 times. The electrodeposited samples are presented on the top row and the spin-coated ones on the bottom row. J5T300, J5T600, and J5T1200 in (**a**–**c**), respectively. SC2000, SC1000, and SC500 in (**d**–**f**), respectively.

The sensitivity of potentiometric sensors depends on the surface morphology of the samples used in the sensing stage of the device, which has been extensively studied for PANI films [41]. A higher sample’s surface area, correlated to its surface roughness, increases potentiometric sensors’ response by increasing the surface sites and, consequently, the charge at the electrolyte/film interface. The surface morphology of the PANI thin films was investigated by means of SEM, presented in Figure 2. The electrodeposited samples are shown on the top row: J5T300, J5T600, and J5T1200 in Figure 2a–c, respectively. The spin-coated samples are shown on the bottom row: SC2000, SC1000, and SC500 in Figure 2d–f, respectively. The surface morphology of the PANI films depends on the deposition method. The electrodeposited films have a granular structure, and the number of nucleation sites increases with the deposition time, as discussed elsewhere [42]. The spin-coated films have a smooth continuous morphology. The appearance of polymeric clusters increases with decreasing angular velocity, as the centrifugal and sheer force decrease and are insufficient to remove such parts that may be assigned as non-dispersed PANI in the DMF [43]. Qualitatively, the electrodeposited films presented a higher surface area, with all their nucleation sites, than the spin-coated ones, preliminarily justifying their sensitivities variations.

**Figure 3:**
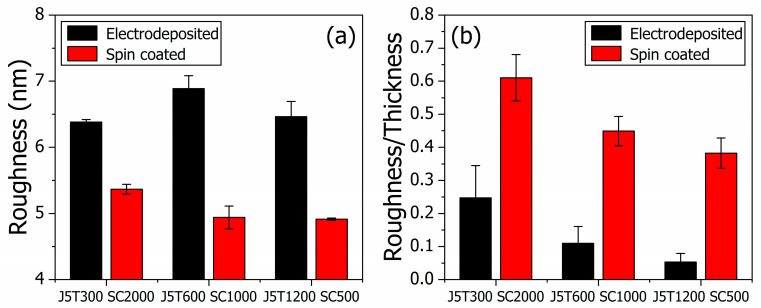
Analysis of the average surface roughness parameters (root-mean-square deviation) in (**a**) and the roughness-to-thickness ratio parameter for both sets of films in (**b**). The black- and red-filled columns show the results for the electrodeposited and spin-coated group of films, respectively.

Quantitatively, the average surface roughness parameters are calculated using root-mean-square deviation and are presented in Figure 3a. Two distinguishable groups indicate that the roughness is higher for electrodeposited samples than for spin-coated ones. The average roughness for electrodeposited films was 6.6 ± 0.3 nm, while for spin-coated films, it was 5.1 ± 0.3 nm. The set of films with a larger surface roughness presented the higher sensitivity, as previously discussed. However, an increase in the surface roughness is also responsible for increasing the charge transfer rate between the electrolyte and sample [44], which would decrease the potentiometric response once the charge transfer process competes with the electric double-layer formation described by a faradaic process [45,46], impacting the potentiometric device. Nevertheless, the sample’s thickness influences the charge transfer rate, and thicker samples have a lower charge transfer rate [47]. The analysis can be conducted by evaluating the roughness-to-thickness ratio (roughness/thickness) to consider the effect of both parameters at once. An enlarged parameter is indicative of a preferable charge transfer process over electric double-layer formation [48]. The roughness/thickness parameter for both sets of films is shown in Figure 3b. There are two distinguishable groups, as a consequence of the same pattern for the roughness of the samples. The average roughness/thickness parameter is 0.1 ± 0.1 for the electrodeposited samples, while it is 0.48 ± 0.12 for the spin-coated ones. The set of films with a larger roughness/thickness parameter presented the lower potentiometric sensitivity, as expected. The decrease in the roughness/thickness parameters is indicative of a larger potentiometric sensitivity, roughly, among each set of films with a specific deposition method. Sample J5T1200, with the higher sensitivity for the electrodeposited films set, presented a lower ratio among electrodeposited samples, 0.05 ± 0.02, while the SC500 sample showed a lower ratio among spin-coated samples 0.38 ± 0.05.

**Figure 4:**
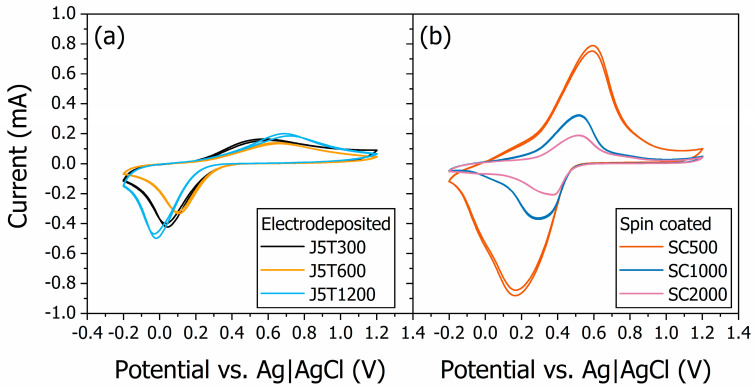
Cyclic voltammetry findings for the electrodeposited (**a**) and spin-coated (**b**) PANI films for the same buffer solution with a pH of 2. The reversible redox peaks of PANI-based materials are present. The peak current depends on the morphological characteristics of the samples determined by the deposition method used for sample fabrication.

The comparison of the cyclic voltammograms (CVs) of the six samples for the same buffer solution with a pH of 2 is depicted in Figure 4. The CVs for the electrodeposited and spin-coated PANI films are given in Figure 4a,b, respectively. The characteristic reversible redox peaks of PANI-based materials are observed in the CVs. Also, the PANI films’ electrochemical response depends on the morphological characteristics of the samples determined by the deposition method. The peak currents (I_Peaks_) for the electrodeposited films were 150 ± 20, 130 ± 10, and 180 ± 20 μA for samples J5T300, J5T600, and J5T1200, respectively. The I_Peaks_ for the spin-coated films were 190 ± 2, 310 ± 20, and 700 ± 80 μA for samples SC2000, SC1000, and SC500, respectively.

The cyclic voltammetry of PANI films is indicative of their redox activity. According to the morphological analysis, the spin-coated samples presented a higher current peak for the CV measurements than the electrodeposited ones, probably due to a favorable electron transfer rate (higher roughness-to-thickness parameter). This also explains the potentiometric sensor behavior of both set of films. Moreover, not only the sample’s morphology, but also the redox state of the polymer are responsible for its electrochemical activity. The ionic current measured via CV is caused by the doping/dedoping of the quinoid rings from PANI [12]. Therefore, the differences in the CVs among the samples are also a consequence of the oxidation state and/or protonation degree of the PANI films. These results agree with those obtained via UV-VIS spectroscopy of PANI thin films. The same is observed in the pH 7.8, where the voltammograms presented differences in the current peak according to the deposition method, but in the μA range (see Figure S4 in the Supplementary Materials).

**Figure 5:**
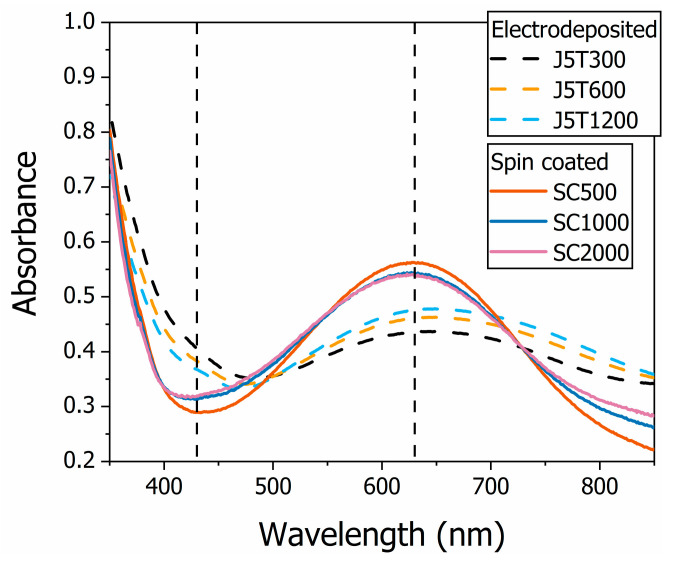
UV-VIS absorption spectra of all the PANI thin films used in the potentiometric pH sensor in this work. The thin films’ absorbance changes due to variations in their oxidation state controlled by the deposition method.

The experimental UV-VIS absorption spectroscopy findings are presented in Figure 5. The relative absorbance is indicative of the polymer oxidation state. A broad absorption peak in the 500 to 800 nm spectral region is observed in doped, undoped, and oxidized PANI with slightly different positions [35]. Nevertheless, the spectra presented the lower energy band, centered around 630 nm, and the higher energy band, centered around 440 nm, ascribed to the exciton transition in undoped PANI and the polaronic transition in oxidized PANI, respectively, characteristic of mixed oxidation state samples [49]. The electrodeposited PANI films presented higher intensity in the low energy band and lower intensity in the higher energy band compared to the spin-coated films. This is an indication that the electrodeposited films are more oxidized than the spin-coated ones [50] and might be related to the galvanostatic oxidation process occurring during electropolymerization [51]. The differences in the oxidation state of the PANI samples reinforce their distinguishable behavior as potentiometric pH sensors. However, all samples presented a change in their spectra after pH sensing to a protonated behavior due to protonation of the quinoid rings, indicating the proton sensing of PANI thin films (see Figure S5 in the Supplementary Materials).

All presented results aimed to determine the effect of the PANI deposition method on the performance of a potentiometric pH sensor. Depending on the deposition method, electrodeposition or spin coating, the structure–property relationship of the PANI thin film changed and affected the sensor’s performance. These are helpful results that also match those observed in earlier studies [20,52,53]. One can control the outcome from potentiometric pH sensors by controlling the deposition process of the chemically sensitive PANI-based thin films.

## 4. Conclusions

The present study was designed to evaluate the effect of the deposition method on the structure–property relationship of chemically sensitive PANI thin films and, as a consequence, on the performance of a potentiometric pH sensor based on them. The samples were characterized by means of their morphology, voltammetry, and UV-VIS spectroscopy and applied as a working electrode on a conventional three-electrode cell, which measured the open circuit potential of the system with buffer solutions of different pH. Electrodeposited and spin-coated PANI films were produced with different deposition times and angular velocities, respectively. The samples were used as the sensing stage of the devices. The electrodeposited films presented an average sensitivity 19% larger than the spin-coated ones (73.4 ± 1.3 mV/pH vs. 59.3 ± 2.5 mV/pH). Among each set of films, the thicker sample presented the higher sensitivity: 74.2 ± 2.2 mV/pH for J5T1200 and 61.9 ± 2.7 mV/pH for SC500. The morphology of the films explains the higher sensitivity for electrodeposited samples. Electrodeposited films presented a rougher surface than the spin-coated ones (6.6 ± 0.3 vs. 5.1 ± 0.3 nm, respectively), which is correlated to a higher surface area and, ultimately, to a higher potentiometric response. The results were supported by the CV analysis that showed a higher electron transfer rate for spin-coated samples, which is a consequence not only of the sample’s morphology, but also of its redox state. Electrodeposited films were more oxidized than spin-coated ones, as presented by the CV analysis and confirmed by the UV-VIS spectroscopy once they presented a relatively higher intensity in the low-energy band (around 630 nm) and lower intensity in the higher-energy band (around 440 nm). Due to the complementarity of the processes, more oxidized PANI films have a decreased redox activity, but an elevated potentiometric response, justifying the higher sensitivity for electrodeposited PANI films. Finally, this study has shown that the sensor’s sensitivity depends on the polymer’s morphology and oxidation state, which rely on the deposition parameters.

## Figures and Tables

**Table 1:** The thickness of the electrodeposited and spin-coated PANI thin films.

Electrodeposited		Spin Coated	
Sample	Thickness (nm)	Sample	Thickness (nm)
J5T300	25.8 ± 9.9	SC2000	8.8 ± 1.0
J5T600	62.9 ± 8.7	SC1000	11.0 ± 1.1
J5T1200	121.5 ± 27.9	SC500	13.2 ± 1.1

**Table 2:** Comparison of sensor’s parameters for the determination of pH for the devices presented in this work and from the literature.

Material	Sensitivity (mV/pH)	Deposition	Correlation Coefficient	Substrate	Ref.
PPY	48	Electrodeposition	0.997	Pt	[36]
PPPD	34	Electrodeposition	0.995	Pt	[36]
PANI	52	Electrodeposition	0.957	Pt	[36]
IrO2	69.9 ± 2.2	Electrodeposition	0.997	polyimide	[37]
WO3 NP	56.7 ± 1.3	Electrodeposition	0.995	polyimide	[38]
TiO2	59	Reactive sputtering	0.998	SiO2/p-Si	[39]
Si3N4	49.7	LPCVD	0.997	Si wafer	[40]
PANI	74.2 ± 2.2	Electrodeposition	0.992	FTO	This work
PANI	61.9 ± 2.7	Spin-coating	0.987	FTO	This work

## Data Availability

The data presented in this study are available on request from the corresponding author.

## Supplementary Materials

The following supporting information can be downloaded at: https://www.mdpi.com/article/10.3390/polym15163450/s1, Figure S1: The sensing PANI films. The top row shows the spin coated samples, and the bottom row shows the electrodeposited samples, respectively. The samples are SC2000 in (a), SC1000 in (b), SC500 in (c), J5T300 in (d), JET600 in (e) and J5T1200 in (f). The red dot squares indicate the region where the UV-Vis spectra were acquired. Figure S2. Graphical analysis to evaluate the conditions of linearity regression concerning the sensors utilizing the J5T600 and SC1000 samples. For the electrodeposited sample, the calibration curve with linear regression in pH range from 2.2 to 7.0 is shown in (a) and the histograms of residuals characterized by a normal distribution in (b) followed by the plot of residuals against fitted values with no trend in (c). The same analysis with the same conclusions is shown for sample SC1000 in (d–f). The Shapiro-Wilk test with *p*-values of 0.972 and 0.967 for J5T600 and SC1000, respectively, rules out non-normality for both sensors indicating that the requisite conditions for linear regression assumptions are confirmed. Figure S3. Chronopotentiometric curves for PANI thin films produce by galvanostatic electrodeposition over the FTO substrate. Figure S4. Cyclic voltammetry for the electrodeposited (a) and spin coated (b) PANI films for the same buffer solution with a pH of 7.8. Figure S5. UV-VIS spectra of the electrodeposited and spin coated PANI thin films before (b) and after (a) pH sensing measurements. References [54,55,56] have been cited in the supplementary materials.
