# Supplementary material for: Understanding the Effect of Deposition Technique on the Structure–Property Relationship of Polyaniline Thin Films Applied in Potentiometric pH Sensor
Source: Polymers (Basel). 2023 Aug 18;15(16):3450. doi: 10.3390/polym15163450 (PMC10459526; doi:10.3390/polym15163450)
Supplement: Supplementary file 1 [file polymers-15-03450-s001.zip › polymers-2500956-supplementary.pdf]

polymers

MDPI

Supporting Information

Understanding the Effect of Deposition Technique on the Structure–Property Relationship of Polyaniline Thin Films Applied in Potentiometric pH Sensor

Vinicius M. Fraga [1], Isabela T. Lovi [1], Luis M. G. Abegão [2,*] and Hugo J. N. P. D. Mello [1,*]

[1] Materials Physics Group, Physics Institute, Goiás Federal University, Samambaia Campus, Goiânia 74001-970, GO, Brazil; viniciusmota@discente.ufg.br (V.M.F.); isabelatalon@discente.ufg.br (I.T.L.)
[2] Photonics Group, Physics Institute, Goiás Federal University, Samambaia Campus, Goiânia 74001-970, GO, Brazil
* Correspondence: luis.abegao@ufg.br (L.M.G.A.); hugomello@ufg.br (H.J.N.P.D.M.)

1. Picture of the PANI thin films

The PANI thin sensing films were deposited by electrodeposition and spin coating. Electrodeposition occurred in an AUTOLAB potentiostat (Metrohm, Herisau, Switzerland) in an aqueous polymerization solution with 0.1 mol/L aniline and 1.0 mol/L HCl. A conventional three-electrode electrochemical cell system was used. The FTO substrate was used as working electrode. The reference electrode was an Ag|AgCl (3 mol/L) electrode, and a platinum foil was used as the counter electrode. The spin coating was performed at 1000 rpm, for 60 s, using a spin-coater (G3P-8 SCS) from a weight ratio of 1:100 polymer:solvent (PANI:DMF, N,N-Dimethylformamide, 99 %, Sigma-Aldrich) solution. Both deposition process occurred over FTO substrates.

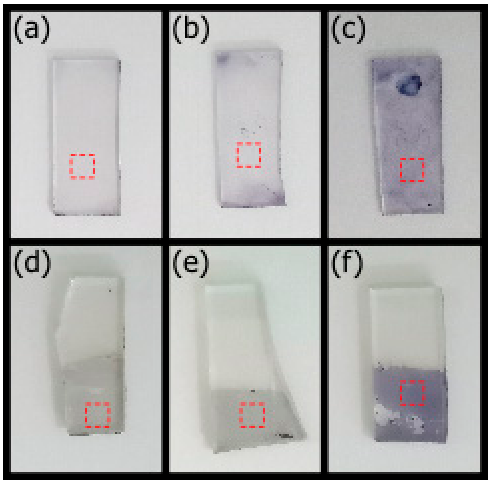

Figure S1. The sensing PANI films. The top row shows the spin coated samples, and the bottom row shows the electrodeposited samples, respectively. The samples are SC2000 in (a), SC1000 in (b), SC500 in (c), J5T300 in (d), JET600 in (e) and J5T1200 in (f). The red dot squares indicate the region where the UV-Vis spectra were acquired.

2. Conditions for sensor's linear regression

Figure S2 illustrates the graphical analysis conducted to assess the conditions of linearity regression concerning the sensors utilizing the J5T600 and SC1000 samples. The analysis of the residuals from the linear regressions serves as a validation for the linear assumption. Figure S-2 (a) and (d) show the linear fit of the sensor's data for samples J5T600 and SC1000, respectively. The linear range for sample J5T600 is from pH 2.2 to 7.0, whereas for sample SC1000, it is from pH 2.2 to 7.8. Errors terms should be normally

distributed in order to apply a linear regression to the data [1], in which Figures S2 (b) and (e) depict the histograms of residuals characterized by a normal distribution for both sample J5T600 and SC1000, respectively. Furthermore, the plot of residuals against fitted values should show no trend [2,3], in which Figures S2 (c) and (f) show that no trend is observed for such plot for ample J5T600 and SC1000, respectively. Given the small sample sizes of the sensors, a Shapiro-Wilk test was executed [2]. This test effectively rules out non-normality for both sensors (p-values of 0.972 and 0.967 for J5T600 and SC1000, respectively). In essence, the data was found to be significantly drawn from a normally distributed population at the 0.05 significance level, thereby affirming the requisite conditions for linear regression assumptions.

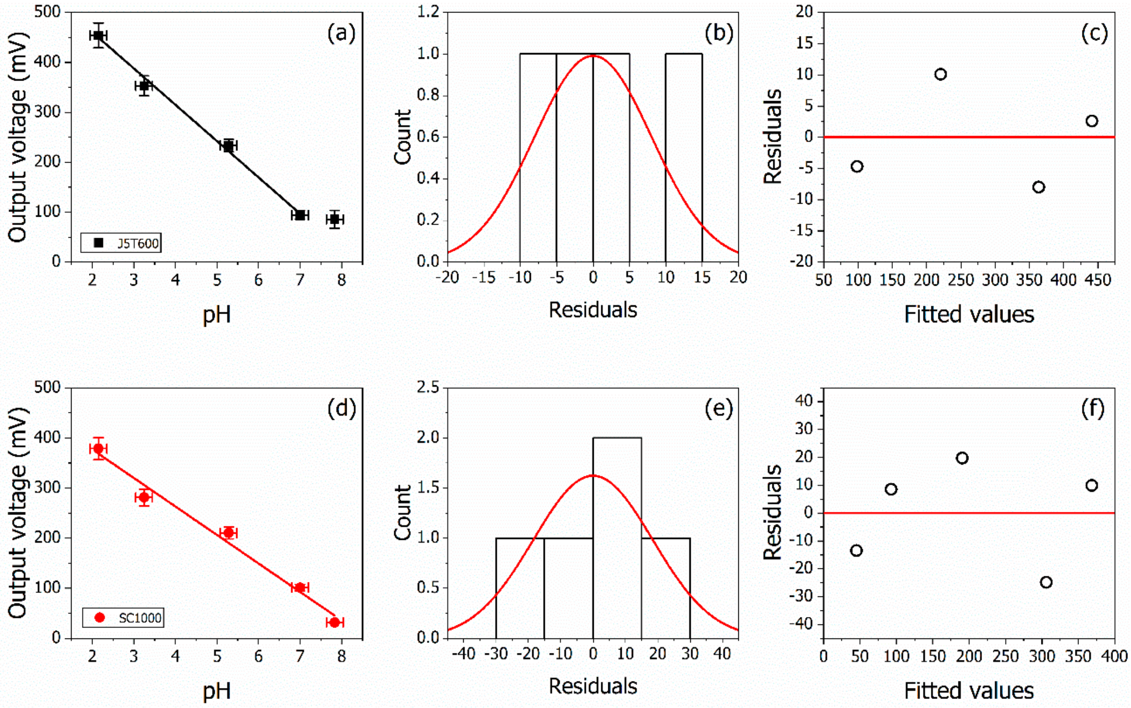

Figure S2. Graphical analysis to evaluate the conditions of linearity regression concerning the sensors utilizing the J5T600 and SC1000 samples. For the electrodeposited sample, the calibration curve with linear regression in pH range from 2.2 to 7.0 is shown in (**a**) and the histograms of residuals characterized by a normal distribution in (**b**) followed by the plot of residuals against fitted values with no trend in (**c**). The same analysis with the same conclusions is shown for sample SC1000 in (**d**–**f**). The Shapiro-Wilk test with *p*-values of 0.972 and 0.967 for J5T600 and SC1000, respectively, rules out non-normality for both sensors indicating that the requisite conditions for linear regression assumptions are confirmed.

3. Chronopotentiometric curves of the PANI thin films

The characterization of the samples regarding their electrodeposition process is shown in Figure S3. The process was described in Figure S-1. The J5T300, J5T600 and J5T1200 samples were electrodeposited with an applied current set as 0.75 mA, for a current density of 0.5 mA/cm^2 during 300, 600 and 1200 s, respectively.

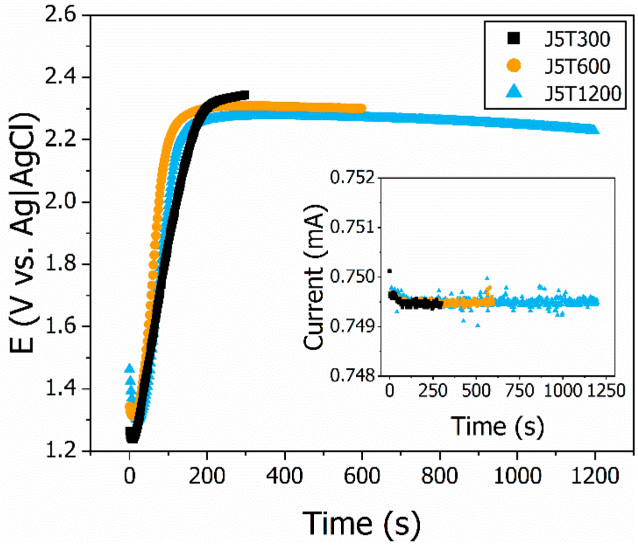

Figure S3. Chronopotentiometric curves for PANI thin films produce by galvanostatic electrodeposition over the FTO substrate.

4. Cyclic voltammograms of the PANI thin films in pH 7.8

The CV response of the PANI thin sensing films in pH 7.8 were obtained in a conventional three-electrode system. The PANI thin films were the working electrode, the reference electrode was an Ag|AgCl electrode, and a platinum foil was used as the counter electrode. CV was applied with a scan rate of 100 mV/s from – 0.2 to 1.2 V (vs Ag|AgCl). As for the CV in pH 2 in the main text, the CV in pH 7.8 for all samples presented the same behaviour with a variation in the peak intensity. Important to note that the current axis is in the microampere range, 1000x lower than in pH 2.

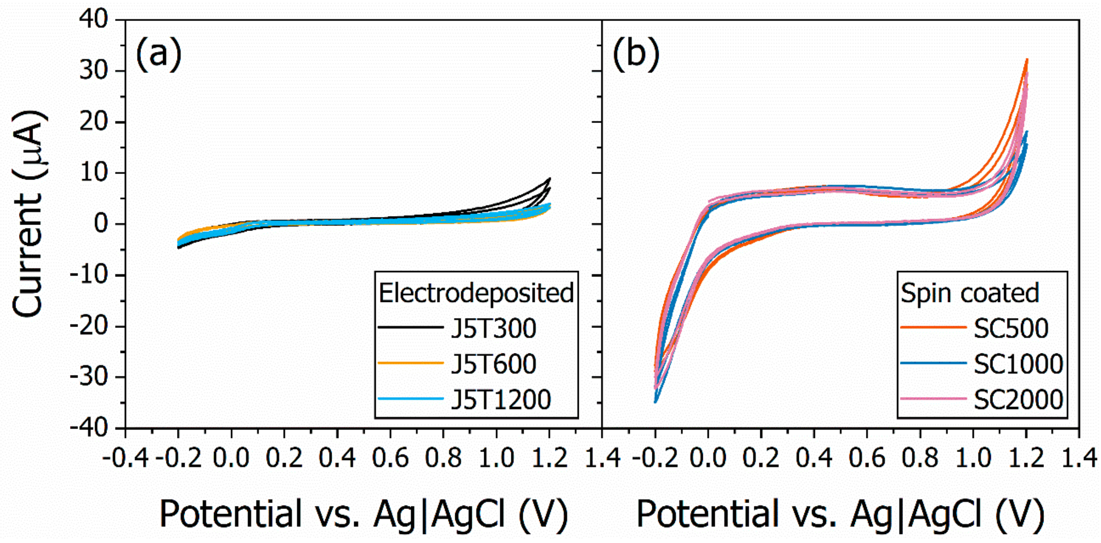

Figure S4. Cyclic voltammetry for the electrodeposited (a) and spin coated (b) PANI films for the same buffer solution with a pH of 7.8.

5. UV-VIS spectroscopy of PANI thin films before and after pH sensing

The UV-VIS optical spectra of the PANI thin sensing films were recorded using a Lambda 1050 WB (PerkinElmer) spectrophotometer in absorbance mode, from 370 to 850 nm in a 0.5 nm step. It is found that the samples after pH sensing presented a protonated pattern, with a shoulder around 440 nm in the absorbance spectra, and before pH sensing they presented an emeraldine base pattern.

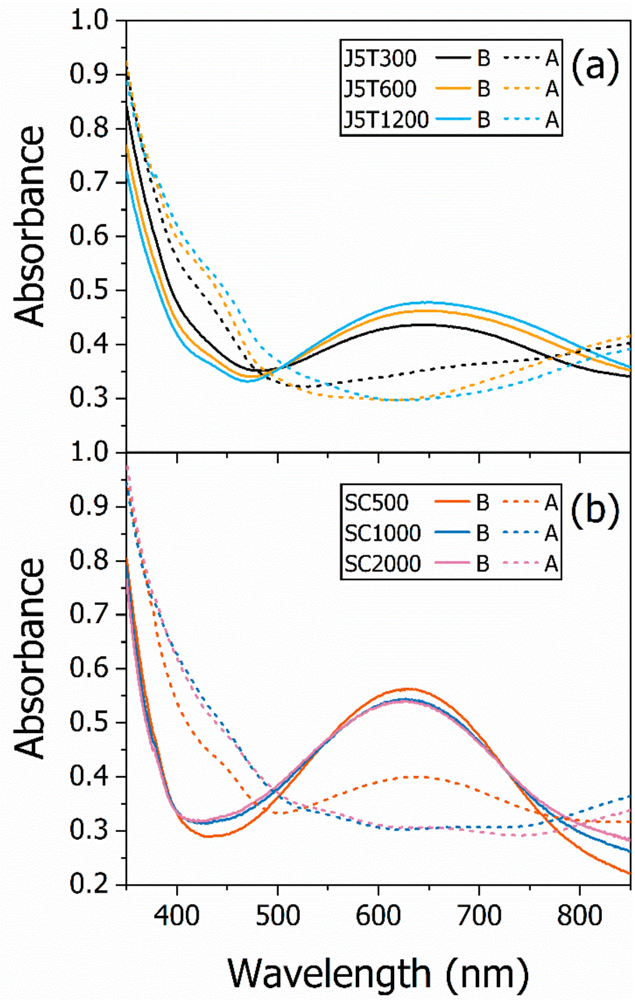

Figure S5. UV-VIS spectra of the electrodeposited and spin coated PANI thin films before (b) and after (a) pH sensing measurements.

References

1. Keith, T.Z. Multiple Regression and Beyond: An Introduction to Multiple Regression and Structural Equation Modeling; 3rd edition.; Routledge: New York, 2019; ISBN 978-1-138-06144-6.
2. Seber, G.A.F.; Lee, A.J. *Linear Regression Analysis*; John Wiley & Sons, 2003; ISBN 978-0-471-41540-4.
3. Schmidt, A.F.; Finan, C. Linear Regression and the Normality Assumption. *J. Clin. Epidemiol.* **2018**, *98*, 146–151, doi:10.1016/j.jclinepi.2017.12.006.
